# Thiamine deficiency impairs common eider (*Somateria mollissima*) reproduction in the field

**DOI:** 10.1038/s41598-017-13884-1

**Published:** 2017-10-31

**Authors:** Torsten Mörner, Tomas Hansson, Le Carlsson, Anna-Lena Berg, Yolanda Ruiz Muñoz, Hanna Gustavsson, Roland Mattsson, Lennart Balk

**Affiliations:** 1 Department of Disease Control and Epidemiology, National Veterinary Institute (SVA), SE-75189, Uppsala, Sweden; 2 Department of Environmental Science and Analytical Chemistry (ACES), Stockholm University, SE-10691, Stockholm, Sweden; 3 Box 140, SE-37222, Ronneby, Sweden; 4 Medical Products Agency, Box 26, SE-75103, Uppsala, Sweden; 5 Department of Biochemistry, Genetics and Immunology, University of Vigo, Lagoas-Marcosende, ES-36310, Vigo, Spain; 6 Vretenvägen 6, SE-75756, Uppsala, Sweden

## Abstract

The Baltic Sea population of the common eider (*Somateria mollissima*) has declined dramatically during the last two decades. Recently, widespread episodic thiamine (vitamin B_1_) deficiency has been demonstrated in feral birds and suggested to contribute significantly to declining populations. Here we show that the decline of the common eider population in the Baltic Sea is paralleled by high mortality of the pulli a few days after hatch, owing to thiamine deficiency and probably also thereby associated abnormal behaviour resulting in high gull predation. An experiment with artificially incubated common eider eggs collected in the field revealed that thiamine treatment of pulli had a therapeutic effect on the thiamine status of the brain and prevented death. The mortality was 53% in untreated specimens, whereas it was only 7% in thiamine treated specimens. Inability to dive was also linked to brain damage typical for thiamine deficiency. Our results demonstrate how thiamine deficiency causes a range of symptoms in the common eider pulli, as well as massive die-offs a few days after hatch, which probably are the major explanation of the recent dramatic population declines.

## Introduction

The Baltic Sea population of the common eider (*Somateria mollissima*) has declined dramatically during the last two decades^1,2^, and the proportion of juveniles wintering in Danish waters has decreased steadily from ca 57% in the early 1980s to only ca 25% in 2009^2^. Recently, widespread episodic thiamine (vitamin B_1_) deficiency has been demonstrated in feral birds and suggested to contribute significantly to declining populations^3,4^. It is well known that thiamine deficiency causes brain damage^5–8^, resulting in neurological disorders and altered behaviour^9–12^, as well as memory and learning disorders^13–17^. Thiamine deficiency as a cause for reproductive disorders impairing recruitment has been addressed in several previous investigations, e.g. in salmonines^18–20^. Despite compelling evidence, however, the debate on the existence of a link between thiamine deficiency and population declines has continued^21^. Here we show that the decline of the common eider population in the Baltic Sea is paralleled by high mortality of the pulli a few days after hatch, owing to thiamine deficiency and probably also thereby associated abnormal behaviour resulting in high gull predation. An experiment with artificially incubated common eider eggs collected in the field revealed that thiamine (T) treatment of pulli had a therapeutic effect on the thiamine status of the brain and prevented death. The mortality was 53% in untreated specimens, whereas it was only 7% in thiamine treated specimens. Inability to dive was also linked to brain damage typical for thiamine deficiency. Our results demonstrate how thiamine deficiency causes a range of symptoms in the common eider pulli, as well as massive die-offs a few days after hatch, which probably are the major explanation of the recent dramatic population declines.

## Results and Discussion

The reproductive output of breeding common eiders was quantified by field inventories in a bird preservation area at the Baltic Sea coast in the County of Blekinge in southern Sweden (Supplementary Fig. 1) 2010–2015. Nests and their number of eggs and/or pulli were counted, as well as females and pulli on the water a few days after hatch. In 2013, common eider eggs from the investigated area were collected for artificial incubation and a thiamine treatment experiment with the hatched pulli. This experiment was motivated by previous investigations in this area 2005^3^ and 2011^4^, where thiamine deficiency was demonstrated in both common eider adults^4^ and pulli^3^, as well as in their major prey, blue mussels^4^.

The common eider has a long breeding season, from mid-April to early June^22,23^. Hence, the variation in the number of active nests (those containing eggs and/or pulli) between inventories a certain year (Supplementary Table 1) was partly due to new nests being built and other nests being left after hatch. The normal clutch size of the common eider is 4–6 eggs per nest^23^. Assuming that the observed clutch sizes (Supplementary Table 1) were a random sub-sample of the population in the investigated area, it was possible to compare the present clutch sizes with corresponding literature values before 1970^24,25^ and thus before the first reports about thiamine deficiency in wildlife. Hildén^24^ observed a mean clutch size for the common eider of 4.6 in the Gulf of Bothnia, Finland, and Coulson^25^ observed mean clutch sizes mostly somewhat higher than 4.6 in Northumberland, UK, before 1970. Hence, the clutch sizes in the present investigation were compared (Z-test) with a value of 4.6 and found to be lower than this value (*P* < 0.01) in all 16 cases with a grand mean of 3.8. This observation indicates that the reproduction of the investigated common eiders is reduced by at least 17%, just by means of fewer laid eggs. Thiamine deficiency has been demonstrated to reduce the number of laid eggs in other bird species, e.g. the herring gull (*Larus argentatus*)^3^. It is thus warranted to suspect that the observed reduced clutch sizes in the present investigation may be due to thiamine deficiency. In Northumberland, the common eider clutch size decreased by ca 25% in the years 1958–1998^25^.

During the inventories we observed very little nest robbing by other birds, such as gulls and crows (*Corvus cornix*). This was also supported by three game cameras monitoring different nests in 2012 and 2013. No monitored nests were robbed, and after hatch, the brood left the nest in the darkness of the night. Inspection of each monitored nest, after the brood had left it, confirmed that the nests had not been robbed and that the eggs had hatched in a normal way. Hence, the low numbers of pulli on the water a few days after hatch (described below) were most probably not due to nest predation.

**Figure 1:**
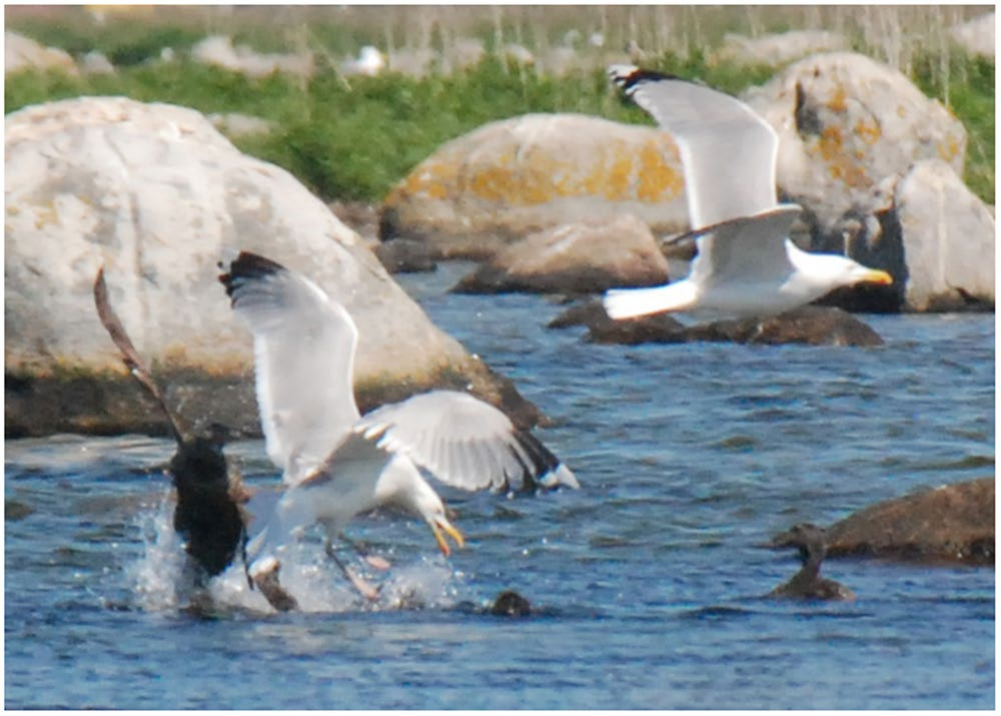
Herring gull (*Larus argentatus*) attack on a common eider (*Somateria mollissima*) gathering at Vållholmen. The pulli neither dived nor ran away, and were thus an easy prey to catch.

In dense breeding areas, common eider broods frequently combine into large gatherings of pulli and females^26–28^, although females may sometimes tend their brood alone^28^. To the best of our knowledge, there is no documentation in the scientific literature indicating any normal occurrence of females without pulli a few days after hatch. Nevertheless, such lonely females dominated in the present investigation. The number of gatherings and their number of component pulli and females, as well as the number of females without pulli, in the investigated area during six years are reported in Supplementary Table 2. Historically, when the environment was less impacted by human activities than today, this area used to host several common eider gatherings with several females and hundreds of pulli each, and no females without pulli. The present situation was the opposite, however, with very few and small gatherings, and hundreds of females without pulli each year (Supplementary Table 2). The number of gatherings a few days after hatch was 2–18 (mean 7), the number of pulli in the gatherings was 8–105 (mean 48), and the number of females without pulli was 61–258 (mean 208). The females without pulli were sitting on the shore or on small rocks, or swimming together in groups of ca 5–15 individuals. On several occasions, we also observed pulli with abnormal behaviour, e.g. when attacked by a gull, the pulli neither dived nor ran away (Fig. 1), and were thus an easy prey to catch^29^. It is also obvious that the number of pulli a few days after hatch was much lower than the number of laid eggs (Supplementary Tables 1 and 2), and the observed number of pulli was only 1–25% (mean 6%) of the (theoretically) expected number of pulli (Supplementary Table 2). These results are indeed disquieting, especially because the same poor breeding result was observed during six consecutive years, i.e. not only temporarily. No bodies of dead pulli where found during the investigations. This observation, in combination with the very low nest predation and the frequent observations of gull attacks on gatherings of females and pulli on the water, indicates that a majority of the pulli were eaten by gulls, and perhaps also other predatory birds, after hatch. Such heavy predation, reducing the number of pulli to few percent within a few days, must be considered abnormal.

The fate of 50 common eider eggs collected at Vållholmen May 21–June 6, 2013, is outlined in Supplementary Fig. 2. A random selection of 37 eggs were allowed to hatch by artificial incubation, whereas the foetuses in the remaining 13 eggs were sampled before hatch and analysed separately. Three pulli were dying during hatch, and were thus excluded from the experiment. The 34 live hatched pulli were randomly allotted to a high T dose group (5 mg T per specimen), a low T dose group (0.5 mg T per specimen), and a control group (carrier or no treatment). The thiamine and carrier were administered by subcutaneous injection in the groin of each specimen. The pulli were observed for 1–5 days with respect to behaviour (including diving) and signs of disease, before they were decapitated, sexed, and sampled for thiamine quantitation in the liver and brain, as well as for histopathological analysis. The 13 foetuses sampled before hatch were sexed. The three pulli that were dying during hatch were sexed and sampled for thiamine analysis.

The sex ratio of the 50 common eider pulli and foetuses is reported in Supplementary Table 3. The null hypothesis of a 1:1 sex ratio could not be rejected (binomial probability test, *P* = 0.065). A balanced sex ratio would be in line with previous investigations of newly hatched common eider pulli^30^. It should also be noted that a balanced sex ratio is an evolutionarily stable strategy, and that differential mortality between the sexes occurring after the period of parental care usually does not affect the neonate sex ratio^31,32^. There was no differential mortality between the sexes in the 37 newly hatched pulli (Supplementary Fig. 3a, P = 1.00).

**Figure 2:**
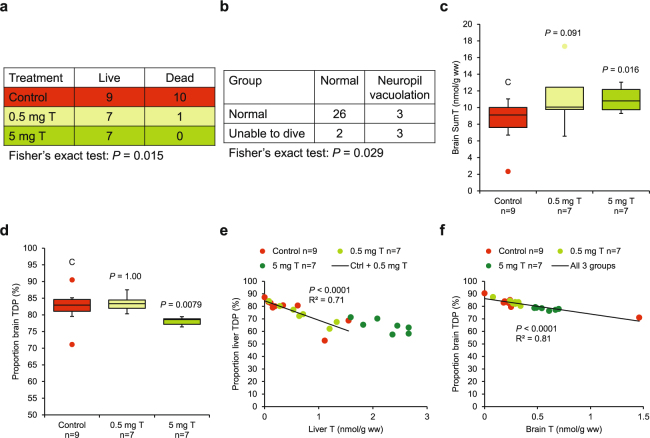
Analytical results of experimental common eider (*Somateria mollissima*) pulli from the County of Blekinge 2013. (**a**) Mortality was low or absent in the two thiamine treatment groups but high in the control group, i.e. death was prevented by thiamine treatment. (**b**) Minimal neuropil vacuolation in the brain was associated with inability to dive. (**c–d**) Thiamine treatment had a therapeutic effect on both brain SumT and proportion brain TDP (the Kruskal-Wallis test, followed by the exact Wilcoxon-Mann-Whitney test as a post-hoc test). (**c**) Brain SumT. (**d**) Proportion brain TDP. (**e–f**) In both liver and brain, there was a negative relationship (ordinary least squares regression) between proportion TDP and T concentration, indicative of various degree of thiamine deficiency^4^. (**e**) Liver. (**f**) Brain. ● Box plots drawn according to Tamhane & Dunlop^48^. C = control.

During the experiment, 11 pulli died: seven in the night (7 control); and four by drowning (1 low dose, 3 control). Thiamine treatment had a positive effect on survival (Fig. 2a, *P* = 0.015). Ten out of 19 untreated specimens (53%) died, whereas only one out of 15 thiamine treated specimens (7%) died. Disablements were observed in 11 specimens and included drowning (1 low dose, 3 control), inability to dive (3 high dose, 1 low dose, 1 control), convulsions (1 control), and weakness (1 low dose). The occurrence of disablements was unrelated to thiamine treatment (Supplementary Fig. 3b, P = 0.69) and mortality (Supplementary Fig. 3c, P = 1.00). It is likely that these kinds of disablements cannot be reversed quickly by thiamine treatment, if at all. It is well known that thiamine deficiency often causes irreversible damage to living organisms, i.e. that physiological conditions may not be fully restored, even though thiamine supply is fully restored. This has been demonstrated for the thiamine-dependent enzymes transketolase^10,33–35^ and α-ketoglutarate dehydrogenase^5,36,37^, as well as for other thiamine-dependent enzymes and metabolites^5,33,37^. Another phenomenon that contributes to irreversible damage is focal cell necrosis, where dead cells are not replaced^8,38–40^. These relatively recent observations that a short-lasting (days–weeks) episode of thiamine deficiency may cause long-lasting (many years or for the rest of an organism’s life) sublethal effects^41,42^ implicates that full recovery by thiamine treatment of afflicted individuals should not always be expected.

No macroscopic lesions were observed in any internal organ in the pulli, and there were no histological lesions in the liver, heart, kidney, lung, pancreas, gizzard, duodenum, thigh muscle, or yolk sac. There were no histological brain lesions in 31 pulli, whereas six pulli displayed minimal neuropil vacuolation in the cerebellar deep nuclei, but no other brain lesions. Pulli with minimal neuropil vacuolation in the brain were over-represented among the pulli that were unable to dive (Fig. 2b, *P* = 0.029). We assume that this behavioural abnormality was directly related to the observed brain lesions. The link between thiamine deficiency, brain damage, and altered behaviour has been demonstrated previously in experiments with birds^4^.

A total of 32 pulli were in medium condition with normally developed muscles and subcutaneous fat, whereas five pulli were in poor condition. Condition was unrelated to the other investigated variables (not shown).

The quantitated forms of thiamine included non-phosphorylated thiamine (T), thiamine monophosphate (TMP), and thiamine diphosphate (TDP). Thiamine concentrations in the liver and brain of the present control pulli were very similar to those in severely thiamine-deficient pulli from partly the same area in the County of Blekinge in 2005^3^ (Supplementary Table 4). Apparently, the thiamine status of the common eider pulli has not improved since the previous investigation. Further indications of thiamine deficiency in the pulli were obtained in the present investigation. Thiamine treatment caused an increase in SumT (T + TMP + TDP) in the brain, at least in the high-dose group (Fig. 2c). This should be impossible if the pulli were non-thiamine deficient from the start, because thiamine treatment should have no therapeutic effect on such individuals^4^. Moreover, thiamine treatment decreased the proportion TDP in the brain (Fig. 2d). This biomarker has been demonstrated^4^ to respond to thiamine deficiency in the following way: At moderate thiamine deficiency (first phase) the cells try to keep as much thiamine as possible as TDP, which is the form active as a cofactor in the thiamine-dependent enzymes. The proportion TDP increases with increasing thiamine deficiency at the expense of the proportions of TMP and T. At more severe thiamine deficiency (second phase), T and TMP cannot decrease any further, and then the proportion TDP begins to decrease with increasing thiamine deficiency. Only the first phase was evident in the relationships between proportion TDP and T concentration in the liver (Fig. 2e) and brain (Fig. 2f). Further aspects of the thiamine injection and analysis are reported in Supplementary Text 1.

## Methods

### Study area

The Blekinge archipelago in southern Sweden was selected as a study area because of previously reported occurrence of thiamine deficiency in the common eider (*Somateria mollissima*)^3^. The investigated islands, situated close to the small town Sölvesborg in the western part of the County of Blekinge, were Vållholmen (56°2′4.4″N, 14°32′14.2″E), Stora Gru (56°1′43.3″N, 14°31′14.2″E), Norra skär (56°1′49.8″N, 14°31′20.6″E), Östra skär (56°1′38.3″N, 14°31′39.6″E), and Söndra skär (56°1′29.7″N, 14°31′30.8″E). The largest of these islands, Vållholmen (0.284 km²), is relatively flat and covered with grass and small shrubberies. It has been used as pasture for cattle and sheep, but is currently not grazed. When this investigation started, the breeding bird fauna was dominated by common eider (ca 250 pairs), herring gull (*Larus argentatus*) (ca 150–200 pairs), barnacle goose (*Branta leucopsis*) (ca 100 pairs), and greylag goose (*Anser anser*) (ca 10 pairs). Other seabird species that breed on the island include mute swan (*Cygnus olor*), shelduck (*Tadorna tadorna*), mallard (*Anas platyrhyncos*), avocet (*Recurvirostra avosetta*), common redshank (*Tringa totanus*), great black-backed gull (*Larus marinus*), common gull (*Larus canus*), black-headed gull (*Chroicocephalus ridibundus*), common tern (*Sterna hirundo*), arctic tern (*Sterna paradisaea*), Caspian tern (*Sterna caspia*), and occasionally also other birds. White-tailed eagle (*Haliaeethus albicilla*) breeds approximately 15 km from Vållholmen, and there are no mammals inhabiting the island. Both white-tailed eagle and mink (*Neovison vison*) visit the island very rarely. Landing on Vållholmen is prohibited during the breeding season, from April 1 to July 15. The other investigated islands, which are situated 0.8–1.0 km southwest of Vållholmen, are of similar character.

### Field studies

Inventories^43^ of common eider nests and their clutch sizes at Vållholmen were performed on nine occasions 2010–2013, and at the other islands on one occasion 2010 (three islands) and one occasion 2011 (four islands). In 2010–2015, 14 inventories were also performed of the number of common eider females and pulli on the water a few days after hatch in the investigated waters outside Sölvesborg. When counting the nests and their clutch sizes, the female flushed from the nest. Only active nests were counted, i.e. nests with eggs and/or pulli. Especially towards the end of the breeding season, many females and their offspring had left their nests. Counted nests were marked with a small colour marker on the grass, or on a stone ca 50 cm from the nest, to ensure that each nest was counted only once. After counting of the eggs and pulli (if any), the nest was carefully covered with down and other nest material to avoid predation before the female returned to the nest. Four nests were excluded from the inventory because they probably contained eggs from more than one female (i.a. >8 eggs according to Cramp *et al*.)^26^. Two nests contained nine eggs each, one nest contained ten eggs, and one nest contained two lighter and two darker eggs. The phenomenon that colony breeding birds lay eggs in each other’s nests is considered abnormal, but has nevertheless been frequently observed in later years e.g.^44^. In 2012 and 2013, three game cameras (Albecom BG529X-8MP, Racerback, Sweden) were used to monitor three separate nests with respect to predation and/or breeding success. In the inventories of females and pulli on the water after hatch, counting was done either from land (2010) or from a small boat going at low speed at a distance of 80–150 m from the shore and following the same route each time (2011–2015).

### Experimental study

In order to study behaviour, condition, disease, sex ratio, and thiamine levels, 50 common eider eggs were collected at Vållholmen May 21–June 6, 2013. Two or three eggs were collected from each one of 21 nests and put on cotton wool in an insulated box and transported to the laboratory within three hours. A random selection of 37 eggs were allowed to hatch in an artificial incubator (R-Com 20 Digital Incubator, Autoelex Co., Ltd., Gyeongsangnam-do, South Korea), whereas the foetuses in the remaining 13 eggs were sampled before hatch and analysed separately. Three pulli were dying during hatch, and were thus excluded from the experiment, although they were sexed and sampled for thiamine analysis. (One of these specimens was, in fact, injected with carrier, but had to be sampled shortly thereafter because it was dying.) The 34 live hatched pulli were kept in the incubator for two to three hours after hatch in order to dry. These specimens were randomly allotted to four experimental groups (Supplementary Fig. 2): 5 mg thiamine per specimen (n = 7); 0.5 mg thiamine per specimen (n = 8); carrier control (n = 7); and untreated control (n = 12). The high and low doses corresponded roughly to 100 mg/kg and 10 mg/kg, respectively. Thiamine (T4625, Sigma-Aldrich, St. Louis, MO, USA) dissolved in physiological saline (0.9% NaCl solution), as well as physiological saline alone, was injected subcutaneously in the groin of each specimen. The injected volume was 0.1 mL. The pulli were then moved to a 35 cm deep pool with a water surface of 1 m² and an adjacent land surface of 0.4 m². Small blue mussels (*Mytilus* sp.), 6–12 mm long, were available *ad libitum* as food on the bottom of the pool. The blue mussels came from natural waters (Baltic Sea) near Mönsterås, Sweden. The pulli were observed for 1–5 days with respect to behaviour (including diving) and signs of disease. The pulli were then decapitated and sampled for thiamine quantitation and histopathological analysis. Those specimens that showed signs of disease or weakness were sampled immediately, whereas specimens that were unable to dive were observed for one day to assure that the inability was permanent. Specimens that died when not attended, and thus were found dead later, were excluded from the quantitation of thiamine. After decapitation, each specimen was weighed to the nearest 1 g. Liver and brain were dissected and weighed to the nearest 0.01 g. The brain was divided along the median plane in a left and a right half. For thiamine quantitation, a liver piece and one half of the brain were put in cryotubes, submerged in liquid nitrogen, and stored at –140 °C until analysis. Samples for histopathological analysis were then dissected from the brain (the other half), liver, heart, kidney, lung, pancreas, gizzard, duodenum, thigh muscle, and yolk sac, and fixed in 10% neutral buffered formalin. Sex was determined in all 50 specimens (both hatched and non-hatched specimens) by the sex dimorphism in the syrinx^45^ and gonads. Non-phosphorylated thiamine (T), thiamine monophosphate (TMP), and thiamine diphosphate (TDP) were quantitated with high performance liquid chromatography (HPLC) with fluorescence detection. The samples were prepared and analysed according to Brown *et al*.^46^ with modifications suggested by Kankaanpää *et al*.^47^ and with the modification that the derivatization reagent, potassium hexacyanoferrate, was prepared in 150 μL of 0.72 M NaOH to a concentration of 0.2%. The chemicals used for the thiamine quantitation are specified by Balk *et al*.^4^. Sample weights were ca 200 mg for the livers and 300–500 mg for the brains. SumT was defined as T + TMP + TDP. Tissue samples for histopathological analysis were dehydrated overnight, embedded in paraffin, and cut in 4 µm sections collected on microscope slides. The sections were stained with haematoxylin and eosin (H&E) and Luxol fast blue, and examined microscopically.

### Data analysis

Because none of the thiamine variables differed (*P* > 0.01) between the carrier control and the untreated control, these two groups were pooled and referred to as just ‘control’. Statistical analysis was made with the softwares Intercooled Stata 12.2 (StataCorp LP, College Station, TX, USA) and R 3.1.0 (The R Foundation for Statistical Computing, Vienna, Austria), and included Fisher’s exact test, the binomial probability test, the Z-test, the Kruskal-Wallis test, and the exact Wilcoxon-Mann-Whitney test. The latter non-parametric tests were used because assumptions of normality and homoscedasticity were not met as analysed with the Shapiro-Wilk normality test and the F-test. Only 2-tailed tests were used. *P*-values below 0.05 were considered significant. Only biological (not technical) replicates were used, i.e. the number of observations corresponds to the number of analysed specimens. In the box plots, the box represents the quartiles: Q1, Q2, and Q3. Two fences are defined as Q1−1.5 × (Q3−Q1) and Q3 + 1.5 × (Q3−Q1). Whiskers are drawn extending from the ends of the box to the most extreme values that are still inside the fences. Observations that fall outside the fences are regarded as possible outliers and are indicated by dots^48^. No statistical calculations were used to determine suitable sample sizes. Instead these were based on general experience of the investigated species. Partial blinding was applied, because complete blinding was not possible.

### Data availability

The dataset, on which the results are based, is available as Supplementary Information.

### Animal care

All use of animals in this investigation was performed in accordance with the permits required by national laws and local regulations. The collection of common eider eggs was approved by the Swedish Environmental Protection Agency, SEPA (Dnr. NV-02953-11) and the Stockholm Northern Research Ethics Committee (Dnr. N58/11). Permission to temporarily visit bird protection areas in the County of Blekinge 2010–2014 was granted by the County Administrative Board of Blekinge (Dnr. 521-441-10).

## Electronic supplementary material

Supplementary Information

Supplementary Dataset
