# Supplementary material for: Thiamine deficiency impairs common eider (Somateria mollissima) reproduction in the field
Source: Sci Rep. 2017 Oct 31;7:14451. doi: 10.1038/s41598-017-13884-1 (PMC5663945; doi:10.1038/s41598-017-13884-1)
Supplement: Supplementary file 1 — Supplementary Information [file 41598_2017_13884_MOESM1_ESM.pdf]

Supplementary Information for

Thiamine deficiency impairs common eider (*Somateria mollissima*) reproduction in the field

Torsten Mörner, Tomas Hansson, Le Carlsson, Anna-Lena Berg, Yolanda Ruiz Muñoz, Hanna Gustavsson, Roland Mattsson & Lennart Balk

**Contents**	**Pages**
Supplementary Figures 1–3	2–4
Supplementary Tables 1–4	5–8
Supplementary Text	9
Supplementary References	10

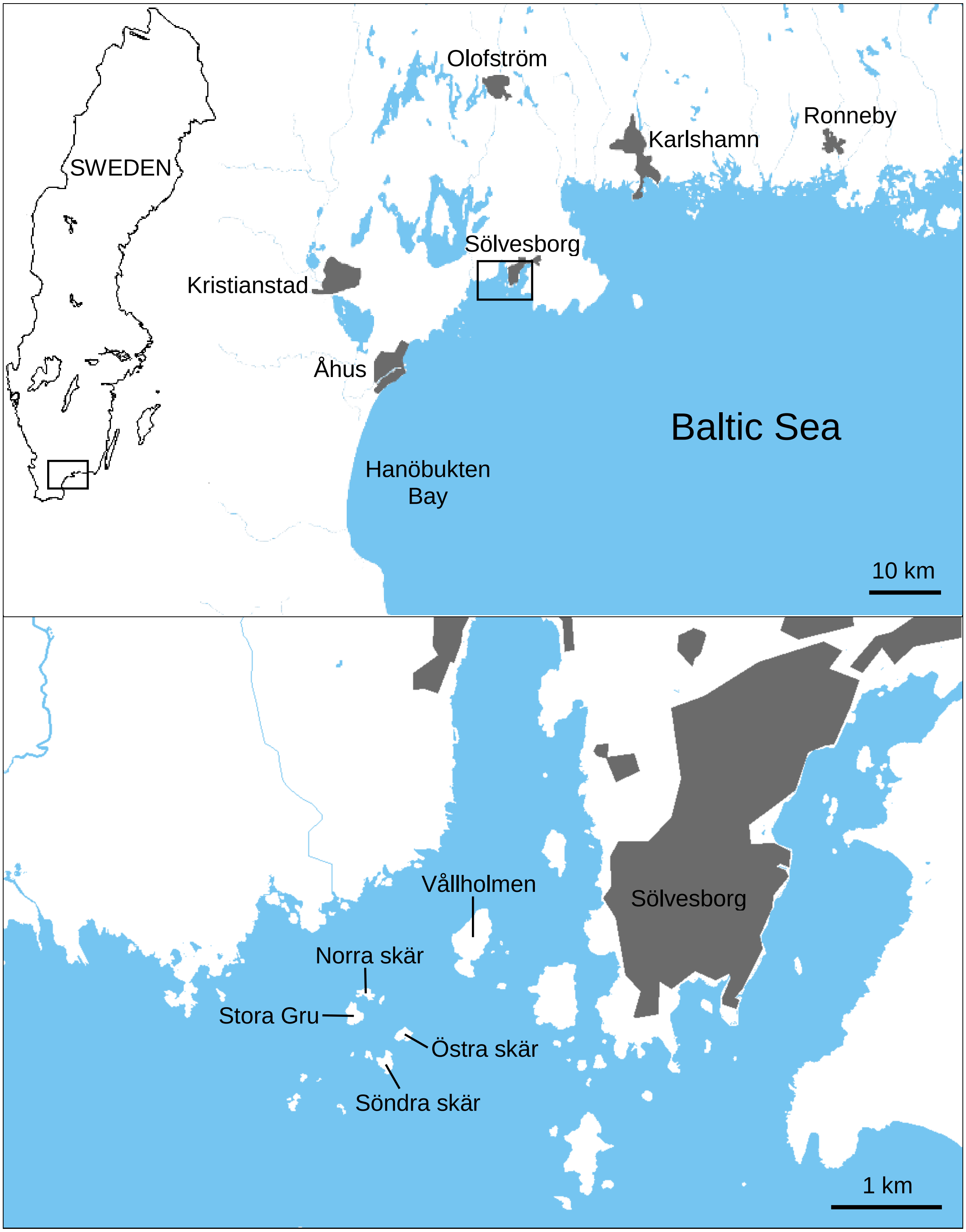

Supplementary Figure 1. The investigated area in the County of Blekinge 2010–2015. The lower map indicates the marine area with the islands Vållholmen, Stora Gru, Norra skär, Östra skär, and Söndra skär outside the small town Sölvesborg in southern Sweden. The maps were created with GIMP 2.8.16 (http://www.gimp.org/downloads/).

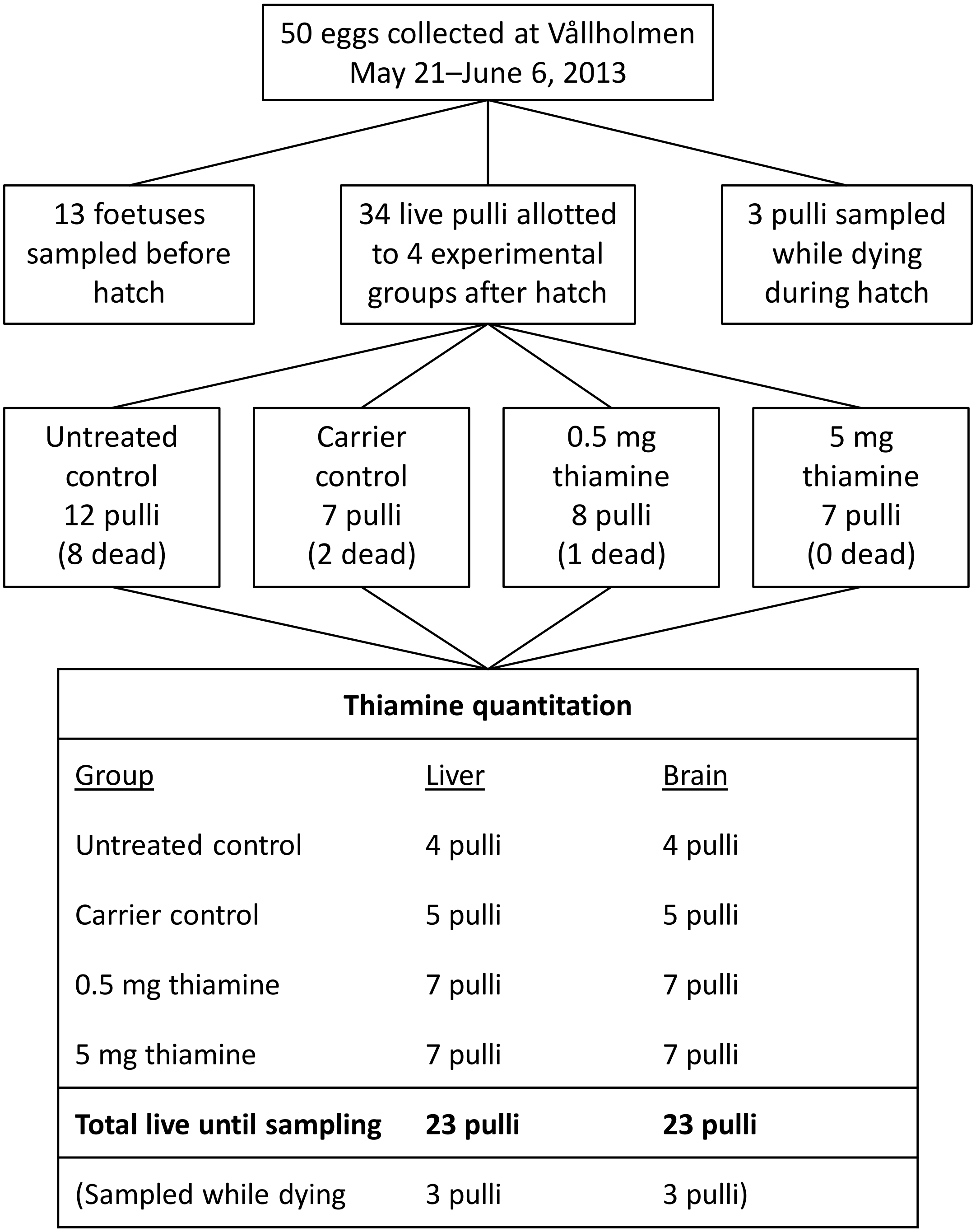

Supplementary Figure 2. Fate of 50 common eider (*Somateria mollissima*) eggs collected at Vållholmen May 21–June 6, 2013. Thiamine was quantitated in live and dying specimens, but not in dead specimens. Dying specimens were unconscious at sampling, but their heart was still beating. The untreated control and the carrier control were pooled and referred to as just 'control'.

a

Group	Live	Dead
Male	9	5
Female	14	9

Fisher's exact test: *P* = 1.00

b

Treatment	Normal	Disable
Control	14	5
0.5 mg T	5	3
5 mg T	4	3

Fisher's exact test: *P* = 0.69

c

Group	Live	Dead
Normal	16	7
Disable	7	4

Fisher's exact test: *P* = 1.00

Supplementary Figure 3. Further analytical results of experimental common eider (*Somateria mollissima*) pulli from the County of Blekinge 2013. (**a**) There was no differential mortality between the sexes. (**b**) Disablements were unaffected by thiamine treatment. (**c**) Disablements were unrelated to mortality.

Supplementary Table 1. Clutch sizes in common eider (*Somateria mollissima*) nests

Place	Date	Nests	Clutch size frequency (eggs and/or pulli)							Sum eggs & pulli	Mean clutch size[a]
			1	2	3	4	5	6	7		
Vållholmen	2010-05-19	135	4	12	18	55	45	1	0	533	3.9
Stora Gru	2010-05-27	30	2	2	6	11	7	2	0	115	3.8
Östra skär	2010-05-27	15	0	0	4	7	4	0	0	60	4.0
Söndra skär	2010-05-27	35	2	5	9	15	4	0	0	119	3.4
Vållholmen	2011-04-21	168	4	9	29	51	65	9	1	699	4.2
Stora Gru	2011-04-25	106	3	8	18	42	31	4	0	420	4.0
Norra skär	2011-04-25	52	4	8	6	15	12	5	2	202	3.9
Östra skär	2011-04-25	78	8	11	18	26	12	2	1	267	3.4
Söndra skär	2011-04-25	88	6	13	26	19	20	4	0	310	3.5
Vållholmen	2011-05-06	100	0	18	18	29	29	6	0	387	3.9
Vållholmen	2011-05-24	97	0	6	19	36	30	3	3	402	4.1
Vållholmen	2012-04-16	59	2	9	17	16	14	1	0	211	3.6
Vållholmen	2012-04-24	122	1	8	23	42	43	4	1	500	4.1
Vållholmen	2012-05-02	76	1	16	16	26	14	3	0	273	3.6
Vållholmen	2012-05-19	33	0	4	7	13	7	2	0	128	3.9
Vållholmen	2013-05-06	144	2	9	19	50	57	7	0	604	4.2

[a] All mean clutch sizes were lower (Z-test, $P < 0.01$) than the literature value 4.6, obtained from investigations performed before 1970[1,2].

Supplementary Table 2. Common eider (*Somateria mollissima*) pulli and females on the water a few days after hatch

Date	Gatherings	Pulli in gatherings	Females in gatherings	Females without pulli	Expected number of pulli[a]	Observed fraction of pulli (%)
2010-05-21	4	52	26	—	—	—
2010-06-02	5	105	36	—	—	—
2010-06-13	5	65	28	—	—	—
2010-06-23	15	95	38	61	376	25
2011-06-05	9	38	22	182	775	5
2011-06-22	18	81	44	218	996	8
2012-05-31	10	50	33	258	1,106	5
2012-06-15	4	10	8	246	965	1
2013-06-05	10	48	28	253	1,068	4
2014-05-30	9	55	48	211	984	6
2014-06-05	2	8	3	227	874	1
2014-06-18	8	39	42	158	760	5
2015-06-05	2	8	4	248	958	1
2015-06-12	3	23	14	224	904	3

— No data.

[a] The expected number of pulli was calculated as the total number of females (females in gatherings + females without pulli) multiplied by an average clutch size of 3.8 eggs per clutch, which is the grand mean clutch size in Supplementary Table 1.

Supplementary Table 3. Sex ratio in 50 common eider (*Somateria mollissima*) pulli and foetuses

**Group**	**Males**	**Females**	**Ratio M/F**	***P*-value[a]**
Hatched pulli	14	23	0.61	0.19
Foetuses	4	9	0.44	0.27
Total	18	32	0.56	0.065

[a] Binomial probability test: in each group, the null hypothesis was a balanced (1:1) sex ratio.

Supplementary Table 4. Thiamine concentrations in common eider (*Somateria mollissima*) pulli in Blekinge[a].

Variable	2005 (n = 16)		2013 (n = 9)	
	Mean	95% CI	Median	95% CI[c]
Liver T (nmol/g ww)[b]	—	—	0.19	0.07–1.07
Liver TMP (nmol/g ww)	0.69	0.44–0.94	1.35	0.73–1.67
Liver TDP (nmol/g ww)	7.2	4.2–10	7.1	4.0–8.6
Liver SumT (nmol/g ww)	8.3	5.0–11	8.6	4.8–10.7
Brain T (nmol/g ww)	0.34	0.27–0.41	0.23	0.18–0.31
Brain TMP (nmol/g ww)	1.1	0.71–1.5	1.24	0.97–1.57
Brain TDP (nmol/g ww)	8.68	5.92–11.4	7.10	5.64–8.47
Brain SumT (nmol/g ww)	10.1	6.94–13.3	9.12	6.84–10.1

— Not quantitated.

[a] The data from 2005 come from Balk et al.[3], whereas the data from 2013 come from the present investigation.

[b] Owing to a chromatographic disturbance, liver T was estimated to 0.4 nmol/g wet weight (half limit of quantification) for all clutches in order to calculate an unbiased value of liver SumT in 2005.

[c] The non-parametric confidence intervals (CI) of the medians 2013 were calculated according to Mood and Graybill[4].

Supplementary Text 1. Further aspects of thiamine injection and analysis.

Time-response patterns of the injection have been considered, but found insignificant for this short duration of the exposure (1–5 days). Possibly, the relatively high liver T concentrations in the high dose group in Fig. 2e (dark green markers) indicate that physiological equilibrium had not been reached after the injection. One low dose specimen had a very high SumT concentration in the liver (53.1 nmol/g) and the brain (17.3 nmol/g). We do not know whether this was an effect of the injection or if this specimen had high SumT concentrations already before the experiment.

It is well known that reliable thiamine quantitation is impossible in dead specimens, and this was confirmed also in the present investigation by thiamine quantitation in liver and brain in some of the dead specimens (not shown, analysed just as a precaution). Substantial dephosphorylation (and probably also thiamine degradation) had occurred in the dead specimens. A new observation was that this process might have started already in some of the specimens sampled while dying during hatch. At least one dying specimen had anomalous proportions of the different forms of thiamine, with a very high T concentration. Hence, a warning should be issued against thiamine quantitation also in dying specimens.

Supplementary references

1. Hildén, O. Ecology of duck populations in the island group of Valassaaret, Gulf of Bothnia. *Ann. Zool. Fenn.* **1,** 153–279 (1964).
2. Coulson, J. C. Variation in clutch size of the common eider: A study based on 41 breeding seasons on Coquet Island, Northumberland, England. *Waterbirds* **22,** 225–238 (1999).
3. Balk, L. *et al*. Wild birds of declining European species are dying from a thiamine deficiency syndrome. *Proc. Natl. Acad. Sci. USA* **106,** 12001–12006 (2009).
4. Mood, A. M. & Graybill, F. A. *Introduction to the theory of statistics. 2nd ed.* 408 (McGraw-Hill, 1963).
